# Analyzing the psychometric properties of the PHQ-9 using item response theory in a Chinese adolescent population

**DOI:** 10.1186/s12991-024-00492-3

**Published:** 2024-01-23

**Authors:** Xuliang Gao, Ziyu Liu

**Affiliations:** School of Psychology, Guizhou Normal University, Huaxi University Town, Guian New District, Guiyang, 550025 Guizhou China

**Keywords:** Item response theory, PHQ-9, Graded response model, Differential item functioning, Psychometric analysis

## Abstract

**Background:**

People are more likely to fall victim to depression during adolescence since it is a period of rapid biopsychosocial transformation. Despite this, most depression research has concentrated on clinical issues, and evaluating depressive symptoms in teenagers is not as widespread. This study used item response theory (IRT) to examine the psychometric properties of the Patient Health Report scale (PHQ-9) in Chinese adolescents. Meanwhile, item function difference tests were used to check whether there were differences in depression symptoms in this group based on education and gender.

**Methods:**

In this research, the PHQ-9 was employed as a measurement tool, and 5958 valid data points were obtained from 12 secondary schools in China (*M*_age_ = 13.484; *SD*_age_ = 1.627; range 11–19 years; 52.17% boys).

**Results:**

IRT shows that all items of the PHQ-9 satisfy monotonicity, unidimensionality and local independence and that they have good psychometric properties. Furthermore, DIF analysis revealed gender and educational disparities in adolescent depressive symptoms.

**Conclusion:**

The study indicates that the PHQ-9 possesses favourable psychometric properties for use in Chinese adolescents. As a result, it serves as a valuable tool for effectively screening depressive symptoms in adolescents. It provides a foundation for prioritizing the development of secondary school students' physical and mental health.

## Introduction

In accordance with the DSM 5-RT, depressive disorders involve the presence of sad, empty, or irritable moods, accompanied by related changes that significantly affect the individual’s capacity to function (e.g., somatic and cognitive changes in major depressive disorder and persistent depressive disorder) [1]. In most cases, however, a variety of physical and mental symptoms, such as smartphone addiction [2], self-harm and suicide attempts in adulthood [3], and eating disorders [4], are also present. The World Health Organisation (2023) states that depression plays a significant role in the global burden of disease and disability. An estimated 3.8% of the population experiences depression, including 5% of adults (4% among men and 6% among women) and 5.7% of adults older than 60 years. Approximately 280 million people in the world have depression. More than 700 000 people die due to suicide every year. Suicide is the fourth leading cause of death in 15–29-year olds [5]. Early childhood and adolescence, which constitute critical neurodevelopmental processes, are the years when depression first manifests. Among other things, mental health vulnerability during adolescence has been supported by research, revealing that almost half of all mental health issues commence before the age of 18 [6]. At the same time, puberty is a dynamic period in which individuals undergo formative changes in their biopsychosocial functioning [7]. It is also the second peak of the self-awareness leap, often characterized by shifts in perspective, increased self-awareness, and personal paranoia. In addition, a meta-analysis has shown that the point prevalence of elevated depressive symptoms among adolescents increased from 24% between 2001 and 2010 to 37% between 2011 and 2020 [8]. Adolescents are in a critical period of development, and they are particularly vulnerable to various social stressors, which might cause many mental health problems [9, 10].

Recent research has explored the factors contributing to adolescent depression in China, particularly in the social and educational domains. On the one hand, China’s rapid transition has resulted in significant changes in life rhythms and ways of thinking, including widening socioeconomic disparity, changes in family structure, and changes in living conditions, all of which have some detrimental consequences on emotions, for example, making the emotional stability of Chinese adolescents decline and generating constant self-denial [11]. On the other hand, given the current state of education in China, various selection exams are fiercely competitive, leading to increased educational competition and risks of over-education and stresses caused by extracurricular tuition and competition for higher education, which negatively impact the mental health of secondary school students [12]. Chinese adolescents' depressive symptoms worsened gradually from grade 10 to grade 12 [13]. Studies have shown that as age increases, the prevalence of depressive symptoms among Chinese secondary school students also increases drastically, from 24.5% in the seventh grade to 40.1% in the twelfth grade [14]. This is because Chinese high school students are under enormous academic pressure as they prepare to take one of the most important exams of their lives: the college entrance exam. The competitive exam in China is described as being like a stampede of ‘one exam for life’.

Research has demonstrated that the importance of evaluation tools is frequently disregarded in practice, which may be one of the reasons for the high rate of undertreatment with antidepressants in routine care [15]. Specifically, with the increased emphasis on mental health, some adolescents may turn to nonprofessionals for psychological counselling or diagnosis or opt for online psychological tests that lack scientific validation, thus leading to increased risks. Nonscientific diagnostic tools may not only lead to diagnostic errors but also generate social exclusion and increase the risk of isolation of the diagnosed person. Therefore, it is imperative to employ scientifically validated diagnostic tools to ascertain the presence of depressive tendencies in adolescents. At present, clinical evaluations and self-assessment scales are two categories of available depression assessment methods. The self-assessment scale evaluates mental health based on actual answers and subjective sensations, allowing the person to recognize their condition and either treat it or work to improve it. The Patient Health Questionnaire-9 (PHQ-9) [16] was initially developed in English for self-administered use in clinical settings in the United States [17]. The PHQ-9 has several advantages over other depression scales. First, the concise format of the PHQ-9 helps improve the attention span of adolescents when responding, especially those who may be more agitated. As a self-report measure, the PHQ-9 allows students to complete the assessment independently, making it easy to capture their subjective experience and provide more comprehensive information. In addition, the questionnaire is designed to be clear and concise with simple language that helps students understand and respond. The PHQ-9 is widely used around the world, has been translated into various languages for research purposes, and has been shown to have great psychometric properties. Examples include Thailand (Cronbach's alpha = 0.79) [18], Uganda (Cronbach's alpha = 0.73) [19], Kenya (Cronbach’s alpha = 0.834) [20], South Africa (Cronbach's alpha = 0.84) [21] and Argentina (Cronbach’s alpha = 0.87) [22]. Based on the above, we chose the PHQ-9 as the measurement tool for this study. Currently, in known studies, the use of the PHQ-9 in China has focused on clinical populations (such as depressed inpatients [23]), college student populations [24], and depression screening in the elderly [25]. There is less literature focusing on adolescents, but two representative papers screened Taiwanese adolescents for major depression [26] or used CTT to analyze depressive symptoms in Chinese adolescents for measurement invariance [27].

With the continuous evolution of measurement theories, it is crucial to explore scales using various measurement frameworks. Conducting cross-validation of scales with different theories not only provides a more comprehensive set of measurement information but also facilitates a deeper investigation into the effectiveness and appropriateness of the scales. For this reason, it is imperative to use item response theory to examine the psychometric properties of the PHQ-9 scale in a Chinese adolescent population. Item response theory (IRT), also known as latent trait theory, is one of the modern psychometric theories and has significant advantages over classical measurement theory (CTT). For example, (a) the IRT allows item responses to be translated into scale-free measures of latent characteristics [28]; (b) the assessment of item-level parameters (e.g., difficulty, discrimination, guessing) is sample independent and has sample invariance [29]; (c) the IRT allows items with functional differences across groups (e.g., gender as well as education as examined in this paper) to be identified [30]; and (d) the standard error of measurement for different participants can be estimated by calculating the amount of information provided by the items at different levels of latent traits.

IRT as a measurement framework involves various latent variable models (commonly used models include logistic models, GRM models, RSM models, GPCM models, etc.), that provide information on psychometric functions at the item and test or scale levels [31]. As a way to assess individuals’ latent traits (ability), represented by $\theta$ (i.e., in this study, the degree of depression), item response functions (IRFs) represent nonlinear associations and the likelihood of a particular response option for the underlying construct [32]. Previous studies have shown that the GRM model is the most appropriate IRT model for ordered polytomous data [33].

Furthermore, depression before puberty is infrequent and affects girls and boys equally. However, as puberty begins, girls are more prone to major depression [34]. Meanwhile, depressive symptoms among Chinese adolescents gradually increase from 10 to 12th grade. As a result, it is crucial to employ the differential item function (DIF), within the framework of IRT to examine whether there are gender and grade differences in depressive symptoms among Chinese adolescents. DIF based on IRT is considered the gold standard for item functional difference testing. It can determine whether there are differences in the responses of subgroups with the same level of actual traits. IRT provides the most comprehensive framework for conceptualizing DIF in polytomous items.

In brief, our study aimed to investigate the applicability of the PHQ-9 in a Chinese teenage population by conducting a more detailed psychometric examination of the PHQ-9 utilizing item response theory with Chinese middle school students.

## Methods

### Procedure

This study received approval from the Institutional Review Board (IRB) of the corresponding author's affiliated institution, ensuring compliance with human research ethics. Written informed consent was obtained from both participants and their parents. The survey was conducted as part of a psychological screening initiative for students by the local schools, and review personnel underwent appropriate training.

Moreover, to ensure the quality of the data, we excluded invalid questionnaires before formal analyses. Data were considered invalid if one or more question response was missing. Based on the above screening criteria, we excluded 78 invalid samples (1.3%) and retained 5958 valid samples.

### Participants

For this study, data were collected in twelve schools in China, including middle school, high school, and secondary vocational schools (students of the secondary vocational-technical school are considered high school students in the following study since they had completed their middle school education). A total of 5958 middle school students completed the PHQ-9, spanning the age range of 11–19 years (in the adolescent stage). The mean age of the participants was 13.484 (SD = 1.627), and the sample included 3109 boys (52.17%) and 2850 girls (47.83%). Of the total sample, 2216 were high school students (37.2%), and 3742 were middle school students (62.8%).

### Measures

Mental health problems were measured by the PHQ-9. The PHQ-9 is a modified version of the PHQ and consists of nine questions. It scores each of the nine symptoms of the DSM-IV criteria according to the frequency of symptoms, namely, the number of symptoms occurring in the two weeks before testing. The scale has four options for each item, ranging from 0 = never to 3 = almost every day, for a total score of 0 to 27.

### Statistical analysis

Statistical analysis was performed using R studio 4.2.2 and IBM SPSS Statistics 29.0 software. The current study had some key goals. First, for normality test: the kurtosis of each item in the scale ranged between 2.220 and 6.711, and the skewness ranged between 1.799 and 2.698. The kurtosis of the total score was 3.381, and the skewness was 1.872. The kurtosis was less than 7, and the skewness was less than 2, indicating that the data were basically normally distributed [35]. Second, the PHQ-9 was assessed to determine whether it met the assumptions of item response theory (unidimensionality, monotonicity, and local independence). Third, the model that best fit the data was selected. Fourth, the functionality of items was assessed using item discrimination, threshold, and item fit. Finally, DIF analyses were used to examine whether there was measurement invariance of depressive symptoms based on PHQ-9 measures across gender and grade levels.

### IRT assumption check

#### Unidimensionality

To explore the robustness of this assumption, we used three methods. A scale was considered one-dimensional if a factor accounted for at least 20% of the variance [36]. Based on this, this study conducted an exploratory factor analysis using the “fa” function in the “psych” package [37] in R to determine whether the scale was unidimensional. In addition, we used the ratio of eigenvalues and confirmatory factor analysis (“lavaan” package) to determine whether the scales were unidimensional [38]. As a rule of thumb, if the ratio of the first eigenvalue to the second eigenvalue is greater than 3, it indicates unidimensionality [39]. The confirmatory factor analysis is guided by the following indicators, namely, the comparative fit index (CFI; ≥ 0.95 for good, ≥ 0.90 for acceptable), the Tucker–Lewis index (TLI; ≥ 0.95 for good, ≥ 0.90 for acceptable), the root mean square error of approximation (RMSEA; ≤ 0.06 for good, ≤ 0.08 for acceptable), and the standardized residual root (SRMR; ≤ 0.06 for good, ≤ 0.08 for acceptable) with its 90% confidence interval.

#### Monotonicity

The monotonicity index is Hi and is interpreted as follows: low quality: 0. 3 < Hi < 0. 40, moderate quality: 0. 40 < Hi < 0. 50, and high quality: Hi < 0. 50 [40]. In our study, the “mokken” package [41] in R software was used to calculate monotonicity with the “check. monotonicity” function.

#### Local Independence

In this paper, local independence is measured in two ways, with Yen's Q3 statistic [42] and Cramer's V statistic. A previous study stated that a value of Q3 above 0.36 suggests moderate deviation and dependence [43]. Cramer’s V is a measure of goodness of fit that determines the independence between variables, and a value below 0.2 indicates independence. For the evaluation of local independence, this paper uses the “residuals” function in the “mirt” package [44].

#### Model fit

IRT differs from CTT in that IRT uses data to fit models and mathematical models to estimate item parameters, participant traits, and other measurement information, while three polytomous IRT models are selected for comparison based on the characteristics of the PHQ-9 in this paper: the generalized partial credit model (GPCM), the rating scale model (RSM) and the graded response model (GRM). Consequently, the “mirt” function is used in the “mirt” package for the Akaike information criterion (AIC), Bayesian information criterion (BIC), Hannan–Quinn criterion (HQ), and likelihood ratio test (LRT) calculations to compare models and to estimate items and individual parameters.

#### Functional assessment of items

The discrimination parameter is an indicator measuring the sensitivity and discriminative power of each item in a measurement tool to the latent traits of the examinees. Threshold parameters play a crucial role in capturing the transition points between response options and indicate the ability levels at which individuals have a 50% probability of shifting from one response option to the next. For example, the first threshold parameter marks the boundary at which participants move from selecting the first response option (e.g., “never”) to choosing the second option (e.g., “several days”). In addition, the fit of the item was tested by $X^2 - RMSEA$, and if the RMSEA was less than 0.6, the item was considered to have good fit. Additionally, we calculated the factor loadings for each item and the amount of information it contained.

#### Differential item functioning

The best-fit model was used as the basis; at the same time, this paper weighed the *p*-value using the “DIF” function from the “mirt” package and effect size examined, with the groupings based on gender and educational year.

In this study, gender and grade differences were examined by calculating the likelihood-ratio test (IRT-LR) for DIF. Due to the large sample size of the study, *p* < 0.001 was selected as the DIF indicator. For each item of the PHQ-9, in the interim, we further checked the effect size by two formulas: $ABS \, ((2 \, * \, (\alpha_g - \alpha_{\text{b}} )/ \, (1.7*\alpha_{\text{g}} \, *\alpha_{\text{b}} )) \, * \, LN \, (2))$ for the discrimination parameter ($\alpha$) and $(\beta ) = \beta_{1b} - \beta_{1{\text{g}}}$ for the threshold parameter. It was considered statistically significant when the effect size was > 0.4.

#### ROC curve analysis

The primary outcome variable is the area under the ROC curve (AUC) [45]. The AUC is interpreted as the probability that a randomly selected respondent will be correctly assigned to the appropriate group [46], directly reflecting the overall accuracy of the instrument in screening for depression. An AUC of 0.5 indicates random performance, while a value of 1 indicates perfect performance. Specifically, values ranging from 0.9 to 1 indicate excellent predictive accuracy, values from 0.8 to 0.9 indicate good accuracy, values from 0.7 to 0.8 indicate fair accuracy, values from 0.6 to 0.7 indicate poor accuracy, and values from 0.5 to 0.6 indicate unacceptably poor accuracy [47, 48].

## Results

### Assumption check

This section assesses the PHQ-9 scale for unidimensionality, monotonicity, and local independence. Unidimensionality analysis reveals a first factor that significantly exceeds the recommended 3:1 criterion for the eigenvalue ratio (7.507:1) used to assess basic unidimensionality [39]. The variance explained in EFA is substantial (0.494), exceeding the threshold of 0.2. Additionally, all indicators performed well in CFA (CFI = 0.981, TLI = 0.974, RMSEA = 0.053, SRMR = 0.021), demonstrating robust unidimensionality of the PHQ-9 scale.

The monotonicity analysis shows moderate monotonicity for all nine items, with values ranging from 0.446 to 0.564, surpassing the 0.4 thresholds. Furthermore, the analysis reveals local independence among item pairs. The Cramer's V values for item pairs range from –0.097 to 0.121, all below 0.2. Similarly, the Q3 statistics for item pairs range from –0.099 to 0.031, all below 0.35. These results suggest that all items on the PHQ-9 scale exhibit local independence.

### Model fit

The lower the value of each model fit statistic, the better the model fits the data. Table 1 shows that the graded response model (GRM) was the optimal model for analyzing the PHQ-9.

**Table 1:** Model comparison

	**AIC**	**BIC**	**HQ**	**LRT**
GPCM	65410.54	65651.47	65494.24	− 32669.27
GRM	64567.75	64808.68	64651.45	− 32247.88
RSM	65950.22	66030.53	65978.12	− 32963.11

*AIC* the Akaike information criterion, *BIC* Bayesian information criterion, *HQ* Hannan–quinn criterion and *LRT* Likelihood ratio tests

## Graded response model parameters

The graded response model has been developed as an extension of the two-parameter logistic model, which is more applicable to polytomous scoring scales. It is worth mentioning that $\theta$ estimates derived using the GRM may show evidence of interval-level scaling properties. The GRM estimates ${\text{k}} - 1$ boundary response functions that represent the cumulative probability of selecting a response option that is better than the option of interest. For example, for the PHQ-9, a scale with four options, analysis with the GRM would have yielded three threshold parameters for each item.

### Item parameters

Table 2 shows the estimated item parameters obtained from the GRM, where all threshold parameters gradually increase (scores were positively correlated with the severity of the participant's depressive symptoms). The differentiation of the PHQ-9 items ranged from 1.927 to 3.456, indicating that all nine items had excellent discriminatory power and were able to distinguish nicely between the presence and absence of depressive symptoms in individuals, of which Item 2 (feeling down, depressed, or hopeless) was the best at discriminating between levels of severity. At the same time, we checked the item fit according to $X^2 - RMSEA$; items showed good fit according to the RMSEA (< 0.06). All items met the criteria and had a good fit. Finally, the factor loadings of all items were between 0.595 and 0.755, more significant than 0.4, indicating that all items measured the ordinal factor (depressive symptoms) well.

**Table 2:** Item parameters, Item fit and Factor loadings

	Discrimination parameter (α)	Threshold parameters (β)			Mean threshold	${\text{X}}^2 - {\text{RMSEA}}$	Loadings
		β_1_	β_2_	β_3_			
Item 1	2.843	0.731	1.459	2.189	1.459	0.009	0.724
Item 2	3.456	0.778	1.444	2.143	1.455	0.008	0.711
Item 3	2.651	0.820	1.490	1.993	1.434	0.008	0.704
Item 4	3.320	0.717	1.426	2.031	1.391	0.007	0.755
Item 5	2.531	0.946	1.658	2.237	1.613	0.012	0.672
Item 6	2.813	0.693	1.362	1.879	1.311	0.008	0.733
Item 7	1.927	0.982	1.722	2.439	1.714	0.009	0.595
Item 8	2.467	1.045	1.688	2.302	1.678	0.006	0.664
Item 9	2.725	1.167	1.701	2.318	1.728	0.012	0.671

$\,\,{\text{X}}^2 .\,{\text{RMSEA}}$ = item-fit statistic; loadings = Factor loadings.

### Information on the items and test

**Fig. 1:**
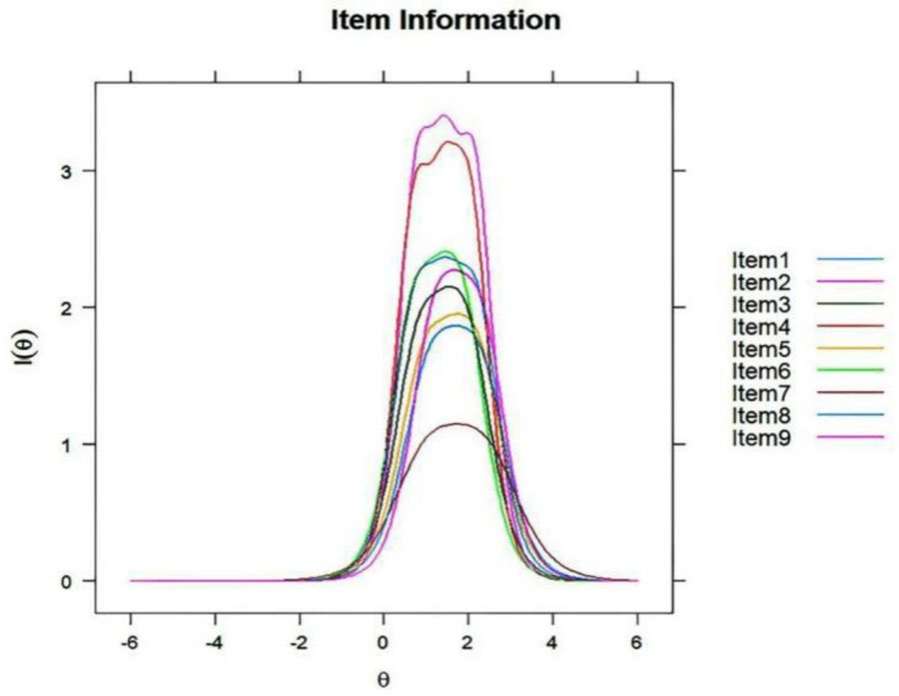
Item information curve

**Fig. 2:**
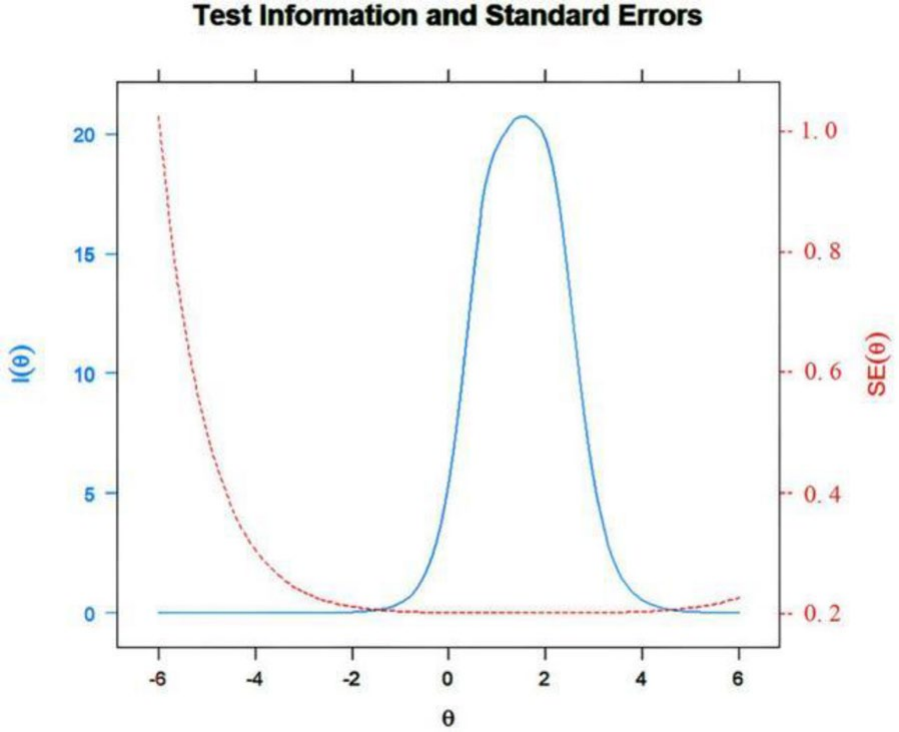
Test information and errors curve

This section presents the item information curves and test information curves. The item information curves, displayed in Fig. 1, demonstrate the relationship between item responses and potential levels of depression ($\theta$). It is evident from the graph that Item 7 has significantly lower information (average = 0.48) than the other items. In contrast, Item 2 provides the highest amount of data (average = 1.27) with lower standard error (SE) values. The test information function (Fig. 2) depicts the levels of $\theta$ at which the scale most precisely and reliably gathers information, which represents the entire range of information the full scale provides, and the peak of the total information curve indicates the level of $\theta$ at which the scale gathers information most precisely. For this particular scale, the test information load peaks when $\theta$ is approximately 2.

In addition, error curves can be observed in Fig. 2. The standard error curve of the measurement shows that the PHQ-9 can provide higher information and lower measurement error when $\theta$ is between 0 and 4. However, the measurement error is higher for participants with lower latent traits. Although the test is more suitable for measuring participants with higher traits, it still demonstrates good measurement errors for participants with lower traits. For instance, with $\theta$ ranging from -4 to 0, the test error was still approximately 0.2. Moreover, considering a normal distribution, the $\theta$ from − 4 to 4 encompasses the majority of the participant population, indicating a high degree of applicability for the test.

### DIF analysis

This section describes the analysis performed to investigate potential differences in depressive symptoms among Chinese adolescents based on gender (boys and girls) and grade (middle and high school), utilizing *p*-values and effect sizes.

### For gender

Table 3 presents the results of the gender difference analysis. There were no significant differences in either *p*-values or effect sizes for the discrimination parameters. However, for the threshold parameters, Items 2 (feeling down, depressed, or hopeless), 3 (trouble falling or staying asleep or sleeping too much), 6 (feeling bad about yourself, that you are a failure, or that you have let yourself or your family down), and 9 (thoughts that you would be better or dead or of hurting yourself in some way) were found to be significantly different. Specifically, 67.82% of girls chose “never” for Item 2, while 80.51% of boys did so. The remaining items with differences were significantly higher for girls than for boys. For Item 6, 7.61% of girls chose “almost every day,” whereas only 3.22% of boys chose this item. These results are consistent with previous findings [34, 49] that suggest that adolescent girls are more susceptible to depressive symptoms than boys. Additionally, girls with major depressive disorder are more likely to experience insomnia, fatigue, psychomotor retardation, and suicide attempts [50].

**Table 3:** DIF for gender (*p*- value & Effect size)

	*p*-value				Effect Size			
	α	β_1_	β_2_	β_3_	α	β_1_	β_2_	β_3_
Item 1	0.058	0.003	0.461	0.546	0.033	0.242	0.222	0.315
Item 2	0.182	0.000	0.000	0.000	0.021	**0.419**	0.344	0.329
Item 3	0.912	0.000	0.000	0.000	0.002	0.318	0.294	**0.458**
Item 4	0.663	0.000	0.002	0.054	0.007	0.286	0.268	0.252
Item 5	0.625	0.000	0.007	0.032	0.010	0.246	0.273	0.299
Item 6	0.072	0.000	0.000	0.000	0.033	**0.479**	**0.477**	**0.556**
Item 7	0.847	0.002	0.012	0.025	0.005	0.174	0.177	0.210
Item 8	0.257	0.017	0.122	0.188	0.025	0.234	0.258	0.319
Item 9	0.155	0.000	0.005	0.326	0.032	0.394	**0.437**	0.361

where bolded data indicate item parameters with significant differences in effect sizes

### For grade

In the analysis of educational differences (Table 4), the comparison between middle school (*N* = 3741) and high school (*N* = 2217) students showed that the differences in the discrimination parameters were not significant across educational levels. This implies that the PHQ-9 is equally valid in measuring the level of depression among participants during their adolescent period. In contrast to the gender differences, the obtained *p*-value and effect size results were completely incoherent for the threshold parameters. Under the *p*-value, there was a significant difference in education on Items 1 (little interest or pleasure in doing things), 2 (feeling down, depressed, or hopeless), 3 (trouble falling or staying asleep or sleeping too much), 4 (feeling tired or having little energy), 5 (poor appetite or overeating), 6 (feeling bad about yourself, that you are a failure, or that you have let yourself or your family down), and 7 (trouble concentrating on things, such as reading the newspaper or watching television). Under the effect size, there was no actual difference. This may be because *p*-values are more likely to be influenced by sample size, which can be applied as a qualitative analysis; effect sizes can be used as a quantitative analysis, as the effect sizes can solve the problem of *p*-values not portraying the magnitude of the correlation and the size of the differences. As in previous studies, children with depressive symptoms had lower average academic performance than their unaffected peers, causing school dropout [51]. This may be related to the Chinese educational model of "thousands of troops crossing a single bridge."

**Table 4:** DIF for grade (*p*-value & Effect size)

	*p*-value				Effect Size			
	α	β_1_	β_2_	β_3_	α	β_1_	β_2_	β_3_
Item 1	0.856	0.000	0.545	0.439	0.004	0.314	0.026	0.112
Item 2	0.593	0.000	0.842	0.199	0.008	0.237	0.033	0.229
Item 3	0.133	0.000	0.018	0.750	0.030	0.254	0.038	0.162
Item 4	0.464	0.000	0.827	0.047	0.012	0.325	0.085	0.115
Item 5	0.053	0.546	0.084	0.000	0.040	0.156	0.064	0.123
Item 6	0.186	0.000	0.164	0.684	0.024	0.269	0.013	0.118
Item 7	0.221	0.000	0.102	0.794	0.034	0.352	0.269	0.167
Item 8	0.125	0.162	0.174	0.123	0.034	0.198	0.056	0.063
Item 9	0.027	0.432	0.715	1.000	0.049	0.134	0.249	0.379

### ROC curve analysis

**Fig. 3:**
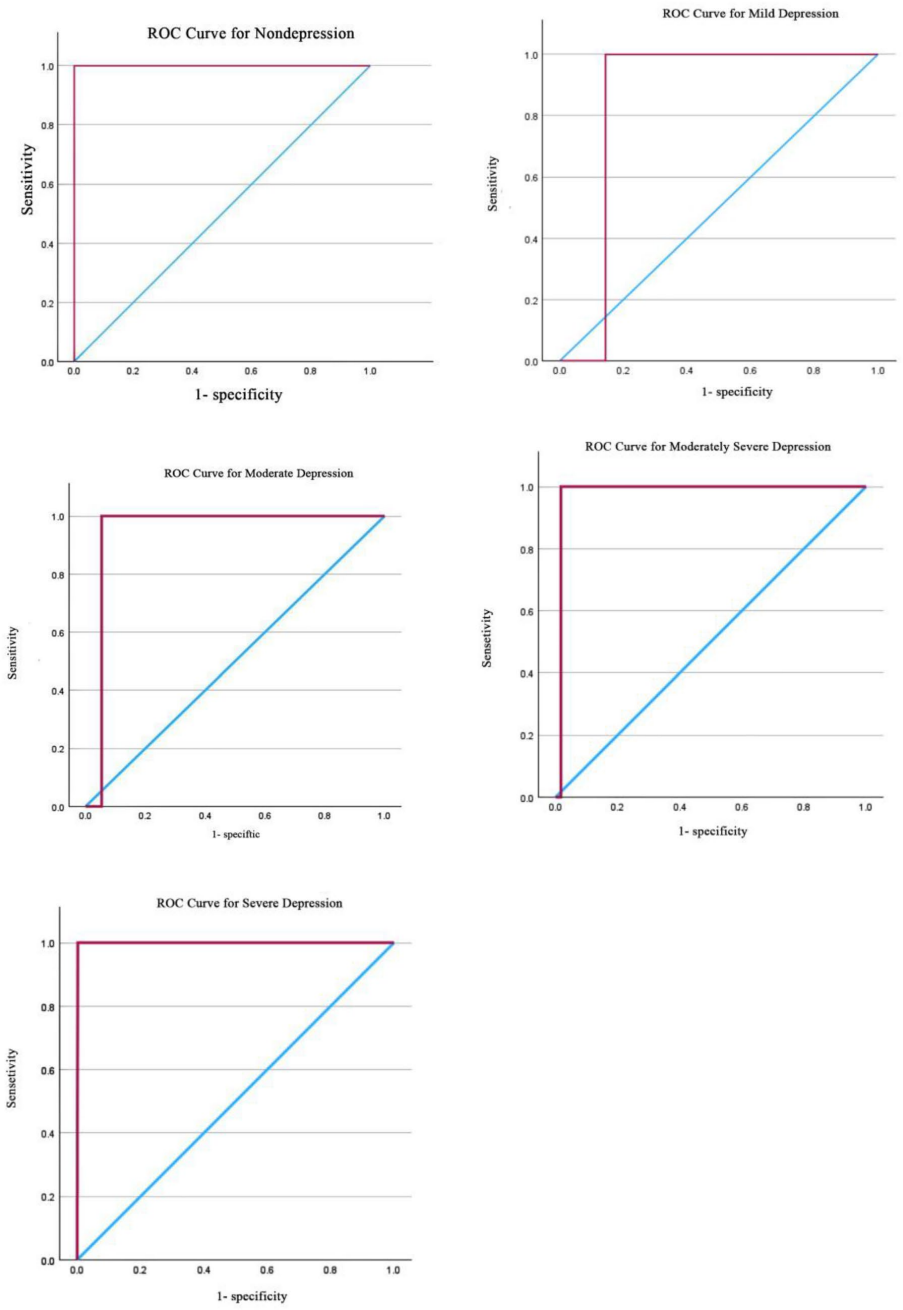
ROC curve for PHQ-9

According to the established diagnostic criteria for the PHQ-9 (the severity of depressive symptoms can be assessed by a total score, i.e., mild: 5–9, moderate: 10–14, moderately severe: 15–19, and severe: ≥ 20 [52]). Receiver operating characteristic (ROC) curves were generated to investigate the applicability of these classification standards in the Chinese adolescent population. Initially, this study plotted five ROC curves, as shown in Fig. 3.

The line of chance is the blue line, and the red line is the ROC curve of the PHQ-9. The closer the curve is to the upper left-hand corner of the graph, the better the diagnostic performance of the PHQ-9. ROC curves were constructed by comparing the nondepressed group with the mildly depressed group; the nondepressed and mildly depressed groups with the moderately depressed group; the nondepressed, mildly depressed, and moderately depressed groups with the moderately severe depressed group; and the nondepressed, mildly depressed, moderately depressed and moderately severely depressed groups with the severe depressed group.

According to Table 5, except for the AUC value of 0.857 for the "mildly depressed" category, all other values exceeded 0.9, indicating excellent performance of the diagnostic criteria. Simultaneously, this study computed the Youden Index through sensitivity and specificity calculations to determine the optimal cut-off ranges for each category (as shown in Table 5). As the PHQ-9 scale yields integer scores, the results align with the original classification standards: "The severity of depressive symptoms can be assessed by the total score, i.e., mild: 5–9, moderate: 10–14, moderately severe: 15–19, and severe: ≥ 20." This classification standard is applicable to the Chinese adolescent population.

**Table 5:** Comparison of ROC curves

	AUC	SE	(95%CI)	Cut-off
Nondepressed	1.000	0.000	1.000–1.000	4.5
Mildly depressed	0.857	0.005	0.847–0.866	4.5
Moderately depressed	0.946	0.003	0.941–0.952	9.5
Moderately severe depressed	0.983	0.002	0.980–0.986	14.5
Severe depressed	0.999	0.000	0.999–1.000	19.5

## Discussion

This study aimed to assess the applicability of the PHQ-9 in a predominantly secondary school student population of Chinese adolescents: the properties and functions of all items were examined, and unidimensional item response theory was used for analysis. IRT is increasingly applied for scale validation, revision, or development. Compared to classical measurement theory (CTT), IRT analysis offers additional advantages, such as presenting a large amount of item-level information that complements CTT more intuitively. However, IRT use must meet the three prerequisites of unidimensionality, monotonicity, and local independence of the scale. Therefore, this study first determined whether the PHQ-9 met the criteria for IRT use. Previous research has confirmed the reliability and validity of the PHQ-9. The PHQ-9 is an internationally accepted depression detection scales, and it contains only nine items. This feature makes it more time-efficient than other scales, and it does not include lengthy questions that may consume the participant's energy and cause distraction, thus resulting in more efficient measurement results. Overall, the PHQ-9 is suitable for IRT analysis and has good psychometric properties. In particular, model comparison analyses reveal that the graded response model (GRM), a commonly used polytomous scoring model, is a good fit for the PHQ-9 data. The current study provides accurate parameter estimates for each item of the PHQ-9 and, through graphic representations (i.e., category characteristic curves, item information curves, and test information curves), provides a visual depiction of the item's function.

In addition, this study conducted DIF analysis and effect size analyses to investigate the differences between the two sets of groups separately. Research on depression and gender differences has widely reported that females are more likely to experience depression than males. These differences are likely due to the reproductive hormones, genetics, environmental variables, and socioeconomic background specific to women's life experiences [53]. Among adolescents with similar latent trait levels, similar results have been found in Chinese secondary students. During individual development, the physical development of males at puberty occurs approximately one year later than that of females [54], and the mismatch between a more tender age and rapid physical development is one of the reasons females are more likely to develop depression. Girls differ considerably more than boys in depressive symptoms such as mood (depression, disappointment), sleep (insomnia, hypersomnia), and self-harm according to the findings of this study. Based on this, future research should explore ways to avoid the current situation where women are more likely to develop depression, such as improving self-identity, improving social status inequalities, and reducing family stress.

Furthermore, the analysis of educational differences revealed a positive association between educational attainment and depressive symptoms among secondary school students. Although the effect sizes did not vary significantly across educational levels, the *p*-values suggested that attention problems, sleep disturbances, and eating disorders increase with grade level. Therefore, providing support and resources for students to manage stress and promote mental health is crucial. These can include a range of interventions, such as counselling services, mindfulness and regimental support, and educational workshops on stress management and mental health, thus improving mental resilience. Home-school associations and a focus on peer interactions help build a healthy environment for children to grow up in.

### Limitations and future research

The findings of this study indicate that as the theta value of the participants decreases, the measurement standard error increases, suggesting that the PHQ-9 scale is more suitable for individuals with higher theta values within the Chinese adolescent population. Additionally, a meta-analysis revealed that COVID-19 not only caused physical health issues but also led to various psychological disorders [55]. Therefore, the results of this study may be influenced collecting data during the pandemic, which could introduce some biases. Further studies should investigate the reliability in more diverse populations and periods to evaluate the scale's validity and generalizability. In addition, the PHQ-9 not only assesses the severity of depression but also has the potential for diagnostic validity. This characteristic makes it valuable for adolescents and responsible parties (such as schools and parents) to identify problems early and intervene to prevent further increase in symptoms. At the same time, there are multiple tools currently available to measure depression. These depression scales should be compared and analysed in the future to investigate the appropriateness of each scale in the context of the community and culture and to assist individuals in selecting the instrument best suited to target individuals to acquire an accurate measurement.

## Conclusions

This study used item response theory as a guide to examine the appropriateness of the PHQ-9 scale in a population of Chinese adolescents. The item functional difference test and effect size analyses were used to examine whether there were differences by gender and educational level. Studies have shown that the PHQ-9 has good psychometric properties among Chinese adolescents. Therefore, the PHQ-9 is an important tool to effectively screen for depressive symptoms in adolescents. Therefore, this study provides a valuable reference for practitioners utilizing the PHQ-9 scale for depression screening in adolescent populations. It not only explores the appropriateness of the scale within the Chinese context but also investigates potential variations across gender and educational levels, adding to our understanding of its practical application and enhancing its utility.

## Data Availability

The dataset used during the current study are available from the corresponding author on reasonable request.

## Abbreviations

IRT: Item response theory
PHQ-9: Patient Health Report scale
GRM: Graded Response Model
GPCM: Generalized partial credit model
RSM: Rating scale model
DIF: Differential item functioning
CTT: Classical measurement theory
IRFs: Item response functions
CFI: The comparative fit index
TLI: Tucker– Lewis index
RMSEA: Root mean square error of approximation
AIC: Akaike information criterion
BIC: Bayesian information criterion
HQ: Hannan–quinn criterion
LRT: Likelihood-ratio test
IRT-LR: Likelihood ratio tests
ROC: Receiver operating characteristic
